# The Diagnosis of Acute Osteomyelitis*Read before the Section on Surgery, State Medical Association of Texas, Galveston, May 11, 1916.— Texas State Journal of Medicine, April, 1917.

**Published:** 1917-07

**Authors:** W. Burton Thorning

**Affiliations:** Houston, Texas


### Journal-Record of Medicine

Successor to Atlanta Medical and Surgical Journal, Established 1855
and Southern Medical Record, Established 1870

OWNED BY THE ATLANTA MEDICAL JOURNAL COMPANY
PUBLISHED MONTHLY

*Official Organ Fulton County Medical Society, State Examing Board, Atlanta, Birmingham and Atlantic Railroad Surgeon's Association, Chattahoochee Valley Medical and Surgical Association, Etc.*

A. W. STIRLING, M. D., C. M., D. P. H.; J. S. HURT, B. Ph.. M.D.
GEO. M. NILES, M. D., W. J. LOVE, M. D., (Ala.); Associate Editors

E. W. ALLEN, Business Manager

COLLABORATORS

W. F. WESTMORELAND, M. D., General Surgery
F. W. McRAE, M. D., Abdominal Surgery
ALLEN H. BUNCE, Medical Societies
E. B. BLOCK, M. D., Diseases of the Nervous System
MICHAEL HOKE, M. D., Orthopedic Surgery
CYRUS W. STRICKLER, M. D., Legal Medicine and Medical Legislation
E. C. DAVIS, A. B., M. D., Obstetrics
E. G. JONES, A. B., M. D., Gynecology
ALLEN H. BUNCE, M. D., Pathology and Bacteriology
R. T. DORSEY, Jr., B. S., M. D., Medicine
L. M. GAINES, A. B., M. D., Internal Medicine
GEO. C. MIZELL, M. D., Diseases of the Stomach and Intestines
L. B. CLARKE, M. D., Pediatrics
EDGAR PAULLIN, M. D., Opsonic Medicine
THEODORE TOEPEL, M. D., Mechano Therapy
E. G. BALLENGER, M. D., Diseases of the Genito-Urinary Organs

VOL. LXIV. ATLANTA, GA, JULY, 1917. NO. 4

### THE DIAGNOSIS OF ACUTE OSTEOMYELITIS.*

By W. Burton Thorning, M.D., F.A.C.S.

Houston, Texas

The following case served to again call my attention to the fact that we frequently fail to recognize acute osteo-myelitis as promptly as we should:

Case History.—A child of five years had been ill, three

*Read before the Section on Surgery, State Medical Association of Texas, Galveston, May 11, 1916.— Texas State Journal of Medicine, April, 1917.

weeks before, with tonsillitis, but had apparently made a good recovery and was playing about as usual.

One week before I saw her she fell from a porch swing and sprained her ankle. The injury appeared to be trivial, as she very quickly resumed her play.

That night she awoke crying with pain in the ankle, which the mother bandaged after applying a liniment. She slept at intervals the remainder of the night, but in the morning she had fever. The ankle was swollen and the pains seemed worse. Accordingly the doctor was summoned. He made a diagnosis of sprained ankle, applied antiphlogistine and gave some Dover's powder for pain.

On his evening visit of the same day, 32 hours after the accident, he found the child acutely ill. Her rectal temperature was 104½ F.; pulse 150; she was delirious, but apparently suffering great pain. The ankle was badly swollen, as was the lower third of the leg, in which region the skin was very much reddened. At this time a diagnosis of acute rheumatism was made and salicylates were administered in addition to the treatment already instituted. On the third day the pain was very much less severe, the temperature had subsided somewhat and the generally improved condition was attributed to the salicylates.

The fourth day she was still more comfortable but complained of a very tender area just above the inner malleous. On more careful examination most of the swelling was found to be at this point, and it was thought fluctation could be detected. Accordingly it was incised and a quantity of pus evacuated. Two days later a skiagram showed extensive necrosis of the tibia, nearly the entire shaft being involved.

I was then called in consultation, but advised that nothing be done, further than to provide adequate drainage for the shaft of the tibia. It will be many weeks, perhaps several months, before the periosteum can form an involucrum to completely surround the sequestrum and before nature separates the dead from living bone.

While contemplating the unfortunate result of a delayed diagnosis in this case it occurred to me that the majority of our diagnostic errors are sins of omission, the results of insufficient study or incomplete physical examinations. The science of medicine is too inexact to exclude errors, but we should strive to make as few as possible and be able to present a reasonable excuse for those we cannot escape.

In looking over my records I was struck by the number of times an acute osteo-myelitis had been improperly diagnosed rheumatism. Our conception of rheumatism has undergone some very radical changes in the past few years, with the result that we no longer consider all acute joint affections as rheumatic in character. In my opinion we should limit our diagnosis of acute rheumatism to those cases which we were formerly taught to consider rheumatic fever; in other words, to the acute infection which results in multiple arthritis, fever, sweats and a tendency to complications like endocarditis, etc., and which is now very generally conceded to be due to streptococcic infection.

The arthritis which occurs as complication of a Neisserian infection has a preceding history and clinical findings that should permit of few errors in its diagnosis. The single joint affections due to other micro-organisms are now looked upon as being due to metastasis from a septic focus perhaps remote.

The chronic affections like arthritis deformans, the various forms of bruritis, etc., are hardly to be confused with acute surgical conditions, hence will not enter into this discussion.

The pathology of acute. suppurative osteomyelitis, as laid down in the text-books, appears somewhat elaborate and complicated, but if we reduce it to its simplest terms and definitely fix in our minds a few points, we need never go far astray in our diagnosis. I do not believe the so-called "idiopathic" or "spontaneous" variety exists, consequently I make the statement that it is always the result of bacterial infection. The organism most frequently present is the staphylococcus pyogenes aures and it is to the type of disease produced by this organism that these remarks apply. Mixed infections are fairly common. We also see occasionally cases where the streptococcus is the prevailing organism and an occasional case due to the colon bacillus, typhoid bacillus and pneumococcus. Very rarely a tuberculous osteo-myelitis has an abrupt beginning, but it is the exception that proves the rule.

If we accept the theory that osteo-myelitis is always hematogenous in origin we must seek the etiologic factors in some primary focus elsewhere in the body. When we are alert in our history taking it is truly surprising how frequently we will find that two or three weeks previously there has been an attack of influenza, tonsillitis, bronchitis, or perhaps an infected ingrowing toe-nail. With such a wide range of possibilities I believe it more rational to assume that we have overlooked a possible source, than to conclude that a given case has occurred spontaneously. We know, as a clinical fact, that sometimes for weeks after certain infections, various microorganisms circulate freely in the blood stream and, unless in overwhelming numbers, produce no definite symptoms. Once they lodge in a medium suitable for their propagation, however, trouble very quickly ensues. Why they so frequently lodge in the epiphyses and metaphyses of long bones is more or less problematical, but I am inclined to accept the theory that it is because of end-arteries in these regions. As an exciting cause we very frequently have exposure or trauma in some degree, only slight, perhaps, but sufficient to lower the resistance in a localized area.

If we accept this theory as a rational explanation of the etiology, it is a perfectly simple matter to build a pathology upon this foundation. If we admit that a septic embolus has lodged in the end-artery of a long bone, what would be the logical, even inevitable, result? A strictly localized abscess which, because of its bony covering, follows the lines of least resistance and rapidly extends through the marrow often the entire length of a long bone, before it can make its way through the pulp canals to the surface and appear beneath the periosteum. The next step in the process is the burrowing of the pus between the bone and the periosteum, elevating the latter, sometimes in a few hours, over a wide area before it perforates into the cellular tissue beneath the skin. The bone thus deprived of its blood supply from both the marrow and periosteum, very quickly becomes necrotic. This, in a few words, it what I believe to be the etiology and pathology of acute suppurative osteo-myelitis and, of a correct interpretation, certainly has the virtue of being easily comprehended. If correct, and clinical observation indicates that it is, we can very readily see why it becomes of the utmost importance to make a correct diagnosis and institute proper treatment at the earliest possible moment.

I once heard the late Maurice Richardson say, that "Any man with a jack knife, an ordinary carpenter's gimlet and the courage of his convictions, can cure any case of acute osteomyelitis." I believe this is literally true, and if we have a reasonably clear conception of the pathology of this condition we can readily see why it should be true. Pus under tension is the pus that does harm and if the tension is relieved further destruction of tissue ceases. As an acute osteo-myelitis is merely a strictly localized abscess with a bony covering, in addition to soft parts, the treatment is comparatively simple.

In differential diagnosis between rheumatism and osteomyelitis we are confronted by almost identical symptoms; pain is usually severe in both, temperature high, tenderness marked and the white blood count increased. In rheumatism we commonly find more than one locality involved, in osteomyelitis it is uncommon. In osteo-myelitis white blood cells average 25,000 to 40,000; in rheumatism the average is lower. In the ordinary case, therefore, a correct diagnosis very largely depends upon the correctness of our observation in physical examination. If all cases of rheumatism presented multiple joint affections there would be little difficulty in recognizing it as such. If all cases of osteo-myelitis began in the middle of the shaft of long bones our mistakes should be few. Unfortunately, however, we find in actual practice that such is not the case. Not infrequently we find only one arthritic joint and not so very rarely do we find multiple foci in osteomyelitis; furthermore, the beginning of the process is almost invariably at one end of a long bone, instead of the middle of the shaft.

The history of the onset of pain is suggestive. In osteomyelitis it is usually abrupt and while it grows steadily worse until it perforates into the cellular tissue, it is usually severe from the first. In rheumatism we often find that the onset is more gradual and in the average case is less intense.

The first and most important point to remember is that in rheumatism the lesion is in the joint; in osteo-myelitis it is not; it is either above or below. In rheumatism inspection shows the tissues around the joint to be puffy and swollen and often reddened; in osteo-myelitis there is no redness and no swelling. (These remarks apply to the early hours of the disease.)

On palpation we find that the capsule of an arthritic joint is tense and very often fluctuation can easily be made out. The point of greatest tenderness is directly over the line which intersects the articulating surfaces. The superficial tenderness is nearly or quite equal to that caused by firm pressure. In osteo-myelitis, in the early hours, palpation reveals nothing; later, when the pus begins to find its way beneath the periosteum, the point of greatest tenderness and swelling will be found, not directly over the joint, but to one side. At this stage the superficial tenderness is slight or absent, but steady, firm pressure, bringing the soft structures into close apposition with the bone, produces a peculiar sickening pain, which is excruciating. Later, when the pus has perforated the periosteum and appeared beneath the skin, palpation reveals the same condition as is found in any superficial abscess.

The pus of an osteo-myelitis sometimes perforates into a joint and produces all the symptoms of a septic arthritis, but it never occurs on the first or the second or third day, consequently, unless the patient delays in calling a physician, it should never occur at all.

Manipulation of an arthritic joint greatly increases the patient's distress; even jarring of the bed is sometimes sufficient to provoke severe spasms of pain. Immobilizing such a joint usually affords relief. In osteo-myelitis slight movements of the adjacent joint do not materially add to the discomfort and immobilization has no favorable influence over the pain.

I do not wish to be understood as laying down hard and fast rules for differentiating these two conditions, as it would be quite impossible to do so. These observations are only general and I am aware that exceptions can be taken to all of them. Neverthless, if they will serve as suggestions only, they may help to make mistakes in diagnosis less frequent.

### DISCUSSION.

Dr. J. E. Thompson, Galveston, said: The diagnosis of this condition is usually very easy. In the large majority of cases, it can be made from the subjective symptoms. In looking over carefully taken histories of cases, one is struck by a similarity in the symptoms as to acute onset, sudden pain and high fever. If added to the subjective symptoms local or objective symptoms, such as tenderness and edema, are present, the diagnosis of osteo-myelitis is certain.

Although the sudden nature of the onset is the rule, one meets with cases of staphylococcic infection of the bone marrow which are more or less chronic from the outset. It is by no means a rare thing to find that the symptoms come on very slowly and insidiously, reaching their maximum at the end of five or six days; but even in these cases, a combination of subjective and objective symptoms will carry us to the right conclusion.

When the diagnosis is made, there should be no procrastination in the matter of treatment. Immediate operation should be undertaken. An incision should be made down to bone over the infected area, and under no circumstances should we stop short of the marrow cavity. Even if pus is found between the periosteum and bone, the operation should be continued until the marrow cavity is thoroughly exposed. Primary, acute, suppurative periositis is a very rare condition, found usually in pyemic conditions, and is not associated with such acute, severe symptoms as acute-myelitis. If operation is undertaken early and the marrow of the bone is exposed it will be found to contain inflamed foci, and occasionally pockets of pus. One should never expect a large pus collection in the early stages. A drop or two of pus will suffice. In many instances, one sees nothing but infiltrated, inflamed bone marrow. In old-standing cases there will be numerous foci present in the marrow.

We have a specimen in the Museum of the University of Texas, which shows the marrow of the shaft of the tibia studded with numerous separate foci from end to end. I have often evacuated three or four separate foci from the marrow cavity of a long bone. In such cases, the whole marrow cavity must be obliterated and a drainage tube inserted into the cavity thus formed. One end of the drainage tube should come out above, near the epiphyseal line or near the epifocus and the other end below at the lowest point of infection.

Dr. P. D. Spohn, Corpus Christi, said: There is no class of cases where delayed diagnosis is more dangerous. Follow the history closely and do not get confused with rheumatism. The disease usually starts abruptly with a rigor, followed by a high fever and severe pain in the limb. The fact that the interarticular portion of the bone is affected, and not the articular, should lead us to differentiate from acute rheumatism. The pain in osteo-myelitis is usually so severe the child screams whenever the limb is touched or the bed moves. The treatment in surgical ab initio, and he who temporizes with fomentations, embrocations and poultices is exposing his patient to untold danger. If you do not find pus the first puncture, repeat until you do find it.

Dr. A. Jacoby, Dallas, asked the author to please state his position in regard to the value of the leucocyte count in the early diagnosis of osteo-myelitis and also in the differential diagnosis between it and acute articular infections.

Dr. T. H. Harrell, Gonzales, said: This paper illustrates a common oversight in surgery. Procrastination in the treatment of osteo-myelitis always means loss of bone and may produce a life long cripple. Heat increases pain in bone; in joint infections it adds relief. Cold relieves pain in bone, but does not in joint infections. It is better to make an exploratory puncture in bone and not find an infection than to let one go undiagnosed.

Dr. Thorning, in closing, said: I am glad that some one called attention to the history of a chill being of diagnostic importance. This, however, is contrary to my experience. Many of my cases have occurred in children too young, and many times too ill, to give a correct history. If a chill had occurred it was overlooked by the parents. I do not argue that a chill is actually rare, but that a history of it is often unobtainable.

In answer to Dr. Jacoby's question, I do not attach any particular significance to the leucocyte count; I regard it only as confirmatory evidence. In my series I am certain the total white count has averaged at least 10,000 higher and the polynuclear percentage from 10 to 15 higher in osteo-myelitis than in acute joint conditions.

My paper was limited strictly to the question of diagnosis and did not include treatment. However, as the question of treatment has arisen in the discussion, I will say that if the diagnosis is made early the treatment is comparatively simple and almost certain of results. In adults it can very well be carried out under local anesthesia; in young children a general anesthetic is generally advisable. An incision is made over the suspected area down to and including the periosteum; the cortex of the bone is then drilled, and if necessary for free drainage a portion is removed. Failure of the pus to immediately appear does not necessarily mean that the diagnosis was incorrect; we may have just missed the pus pocket and a few hours later we may find the dressings saturated with pus and the pain entirely gone.

The point in the treatment to be emphasized in this: If we suspect osteo-myelitis strongly enough to warrant making an incision we are not justified in stopping short of drilling the cortex of the bone.

